# 510. Real-world Effectiveness of Sotrovimab for COVID-19: Evidence from United States (US) Administrative Claims Data

**DOI:** 10.1093/ofid/ofad500.579

**Published:** 2023-11-27

**Authors:** Christopher F Bell, Tasneem Lokhandwala, Daniel C Gibbons, Myriam Drysdale, Jane Wang, Emily Lloyd

**Affiliations:** GlaxoSmithKline, Durham, North Carolina; Xcenda LLC, Carrollton, Texas; GlaxoSmithKline plc., Brentford, Middlesex, UK, Brentford, England, United Kingdom; GSK, Brentford, Middlesex, England, United Kingdom; Xcenda LLC, Carrollton, Texas; GSK, Brentford, Middlesex, England, United Kingdom

## Abstract

**Background:**

This study compared outcomes among COVID-19 patients (pts) treated with the monoclonal antibody (mAb) sotrovimab matched to untreated pts.

**Methods:**

Administrative claims (Komodo Health) were used to identify pts (≥ 12 years) diagnosed with COVID-19 (ICD-10: U0.7.1) in an ambulatory setting in the US (26 May 2021–5 April 2022). The sotrovimab cohort included pts meeting sotrovimab’s Emergency Use Authorization (EUA) criteria and receiving sotrovimab ≤ 10 days from diagnosis (index date = day of infusion). The untreated cohort included pts with no evidence of early/prophylaxis mAb or antiviral treatments (index date imputed based on the time distribution between diagnosis and sotrovimab infusion for their matched counterpart from the sotrovimab cohort). Pts were required to be continuously enrolled for ≥ 12 months pre-index and ≥ 29 days post-index (excluding death). Exact matching and propensity-score matching (EM/PSM) methods were used to construct 1:2 matched cohorts of sotrovimab-treated and untreated patients (EM; age [≤ 5 years], diagnosis date [≤ 14 days], state: PSM; gender, payor, region, Quan-Charlson score, EUA criteria, index date, baseline healthcare utilization). Outcomes evaluated in the 29-day post-index period were all-cause hospitalization, all-cause hospitalization and/or mortality, all-cause mortality, intensive care unit (ICU) admission, and maximum level of respiratory support.

**Results:**

A total of 34,160 sotrovimab pts were matched to 68,320 untreated pts (characteristics well balanced: 54 years of age, approximately 40% male, 42% had commercial payor, and approximately 50% indexed in January 2022). The 29-day hospitalization and/or mortality rates were 4.0% (n=1,380 / 34,160) and 5.2% (n=3,566 / 68,320) in the sotrovimab and untreated cohorts, respectively (p < 0.0001). Patients in the sotrovimab cohort had statistically significantly lower rates of all-cause hospitalization, all-cause mortality, ICU admission, and respiratory support as compared with patients in the untreated cohort (Table).

**Figure:**
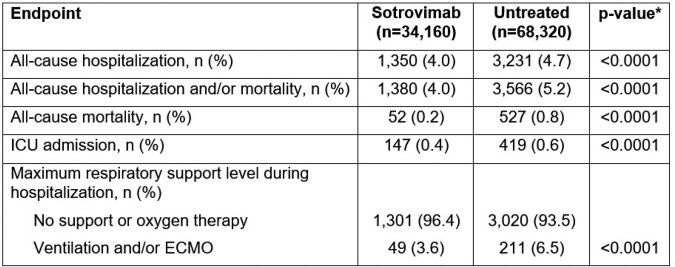

*Obtained from conditional logistic regression models (binary outcomes). All models adjusted for malignancy. ECMO, extracorporeal membrane oxygenation; ICU, intensive care unit

**Conclusion:**

Sotrovimab demonstrated effectiveness in preventing severe outcomes (hospitalization, mortality and respiratory support) as compared with untreated pts during the period in which Delta and early Omicron variants were predominant.

**Disclosures:**

**Christopher F. Bell, MS**, GSK: Employee|GSK: Stocks/Bonds **Tasneem Lokhandwala, PhD**, GSK: Funding to conduct study|Vir Biotechnology, Inc: Funding to conduct study **Daniel C. Gibbons, PhD**, GSK: Employee|GSK: Stocks/Bonds **Myriam Drysdale, PhD**, GSK: Employee|GSK: Stocks/Bonds **Jane Wang, MS**, GSK: Funding to conduct study|Vir Biotechnology, Inc: Funding to conduct study **Emily Lloyd, MSc**, GSK: Employee|GSK: Stocks/Bonds

